# The Use of Si(C,N) Layers as Barrier Coatings in Dentistry

**DOI:** 10.3390/ma19122568

**Published:** 2026-06-14

**Authors:** Zofia Kula, Grzegorz Szparaga, Małgorzata Siatkowska, Leszek Klimek

**Affiliations:** 1 Department of Dental Technology, Medical University of Lodz, 251 Pomorska Street, 92-213 Lodz, Poland; 2 Textile Institute, Lodz University of Technology, 116 Zeromskiego Street, 90-924 Lodz, Poland; grzegorz.szparaga@p.lodz.pl; 3 Department of Biophysics, Institute of Materials Science and Engineering, Lodz University of Technology, Stefanowskiego 1/15, 90-924 Lodz, Poland; malgorzata.siatkowska@p.lodz.pl; 4 Brain Laboratories, Medical University of Lodz, Czechoslowacka 4, 92-216 Lodz, Poland; 5 Institute of Materials Science and Engineering, Lodz University of Technology, 90-924 Lodz, Poland; leszek.klimek@p.lodz.pl

**Keywords:** dental alloy, Si(C,N) coatings, ion release

## Abstract

The corrosion phenomenon can cause negative allergic and cytotoxic reactions in the human body, inflammation, and, in the future, the development of cancer. Their sources may be corrosion products, metal ions released during the corrosion process, and galvanic currents that penetrate the surrounding tissues. In order to avoid the negative effects of using metal alloys, their surface can be modified by applying coatings. The aim of this study is to determine and compare the amount of ion release from Si(C,N) coatings with varying carbon and nitrogen contents, as well as from the uncoated substrate alloy (Group A) in various aqueous environments. Si(C,N) coatings were applied to the surface of the prosthetic alloy. Si(C,N) coatings with different carbon and nitrogen contents were deposited using the reactive magnetron sputtering (RMS) method. The research included determining the amount of ions released into the environment: distilled water, 0.9% NaCl and artificial saliva. Assessments were made at 10, 30 and 90 days. All tested Si(C,N) coatings significantly limit the amount of metal ions in the surrounding medium. Due to the lack of statistically significant differences in the number of ions released by individual coatings, when selecting them, other properties related to the operating conditions of the elements should also be taken into account.

## 1. Introduction

For many years, metal alloys have played an important role in the fields of medicine and dentistry. These materials have found applications in all areas of dentistry. In dental prosthetics, they are most often used as permanent restorations (crowns, bridges) and removable restorations (partial and full dentures) in the casting process [1,2,3]. Prosthetic restorations, as medical products, must meet appropriate requirements due to their location in the living human body. In particular, they must be characterized by high biocompatibility and must not cause allergic or cytotoxic reactions in the patient or the entire dental team (dentist, hygienist and dental technician) [3,4,5]. These alloys must be resistant to corrosion and abrasion in the oral environment and be sufficiently hard and durable, which impacts their subsequent processing and use. Economic considerations are also important (relatively inexpensive and readily available). Metal alloys used for veneering the substructure with ceramics should be resistant to high temperatures [3,4,5,6,7].

Both prosthetic and orthodontic elements placed in the oral cavity are exposed to the aggressive action of the oral environment (temperature differences, pH changes, electrochemical activity, presence of ions, e.g., fluorides). All these factors can influence the initiation of corrosion processes in the metal structure. Some scientific sources indicate that an increase in the concentration of fluoride ions and a decrease in the pH of artificial saliva can significantly reduce the corrosion resistance of the titanium alloy [8]. Factors accelerating the corrosion process may also include an inappropriate diet, taking certain medications or improper oral hygiene [9,10,11].

The corrosion phenomenon can cause negative allergic and cytotoxic reactions in the human body, inflammation, and, in the future, the development of cancer. Their sources may be corrosion products, metal ions released during the corrosion process, and galvanic currents that penetrate the surrounding tissues [10,11,12,13]. Metal ions released during the corrosion process from the surface of dental elements can migrate to the digestive system and then accumulate in the liver, kidneys, spleen, bones, lungs, brain or mucous membrane [12,13]. The presence of ions in the tissues around a dental implant can be a product of electrochemical corrosion and frictional wear. Corrosion products resulting from the interaction of metal with the surrounding oral environment affect the biocompatibility and long-term durability of prosthetic restorations [14].

An allergic reaction is defined as an acquired disorder consisting of hyperreactivity in contact with a foreign allergenic substance and is determined by genetic predisposition. Allergic reactions are most frequently observed among patients when using various elements made of nickel, chromium and cobalt. However, nickel is considered the most allergenic metal of all. It is the cause of contact allergies in the general population worldwide: 11.4% in Europe, 8.8–25.7% in Asia, 17.5% in North America [15,16]. In addition to its high allergenic potential, Ni may also act as an immunotoxic and carcinogenic agent and may contribute to acute and chronic diseases of the circulatory and respiratory systems. The most common symptoms of allergic reactions include skin lesions, eczema, redness, and itching. These observations may apply to the use of both orthopedic and dental implants as well as orthodontic elements [15,16,17,18,19]. Allergic reactions can also cause asthma, conjunctivitis, inflammation of the mucous membranes and produce extraoral and systemic symptoms. In severe cases, anaphylactic shock. They usually resolve immediately after contact with the allergen is removed [17,18]. They can take the form of metallosis, or the localized impact of metal ions and corrosion products from a prosthetic element or implant on human tissue. Long-term contact with the allergen may cause inflammation of the bone or bone marrow without any skin lesions [16,18].

Despite the unfavorable effects of metal alloys, they are still used in dental technology as structural elements of prostheses or in dental implantology [20]. In order to avoid the negative effects of using metal alloys, their surface can be modified by applying coatings. The use of coatings for medical purposes is quite popular. The use of metal carbides, oxides and nitrides has become particularly popular. They are characterized by strength and corrosion resistance. They are used in the medical device industry, for example, in orthopedic prostheses, dental implants, and catheters, as drug-eluting coatings on stents, and as blood-compatible coatings (such as heparin).

Current research focuses on the deposition of Ti(C,N) coatings, particularly for medical applications. In our work, we used Si(C,N) coatings deposited by reactive magnetron sputtering (RMS) on the surface of a prosthetic Ni-Cr alloy. The aim of this study is to determine and compare the amount of ion release from Si(C,N) coatings with varying carbon and nitrogen contents, as well as from uncoated coatings, in various aqueous environments. The null hypothesis is that Si(C,N) coatings cannot affect ion release.

## 2. Materials and Methods

**Figure 1:**
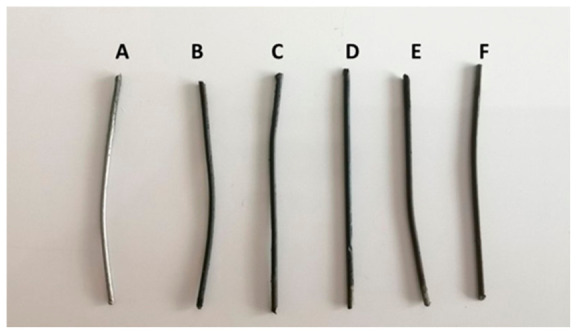
Prepared specimens.

The first stage of sample preparation was the production of wax prototypes in the shape of rods. For this purpose, 2 mm diameter wax shapes from Rentfert (Hilzingen, Germany) were used. The wax samples were attached to the casting ring using 4 mm diameter wax casting channels. One method used in the casting of prosthetic components is the so-called “lost wax method,” which involves pouring an investment material onto wax structures and then burning them to create a mold into which molten metal is introduced. The NiCr Heraenium alloy from Heraeus Kulzer (Hanau, Germany) was used in this work. The cast rods had the following dimensions: 2 mm in diameter and 45 mm in length (Figure 1). Coatings were applied to the prepared samples using magnetron sputtering.

The parameters and course of the deposition process, as well as the chemical composition of the Ni-Cr alloy coatings, were described in detail in previous research papers [21]. The samples were divided into six groups: one group without a sputtered layer and the remaining five groups with Si(C,N) layers with different carbon and nitrogen contents. Table 1 presents the composition and designation of the individual specimens.

Ion release studies were performed in three different environments: distilled water, physiological saline solution and artificial saliva prepared according to the Fusayama-Mayer procedure [9]. Individual groups of metal rod samples were immersed in 30 mL polystyrene tubes. Each tube was filled with 10.0 mL of fluid. In each environment, the samples were immersed for 10, 30 and 90 days at a temperature of 36.7 ± 0.2 °C. For each immersion period, independent samples were utilized to ensure that evaluations at different time points were conducted on separate, distinct specimens. In order to obtain conditions as close as possible to those in the oral cavity, Incubator Shakers were used.

Chromium and nickel concentrations were determined by flame atomic absorption spectroscopy using a Thermo Scientific iCE 3500 AA System spectrometer (Cambridge, UK). Hollow cathode lamps appropriately matched to the elements being determined were used for the measurements. For Cr, a wavelength of 357.9 nm was used as the main analytical line and 354.9 nm as the control line. For Ni, wavelengths of 232.0 nm (main line) and 232.1 nm (control line) were used. Measurements were conducted using an air-acetylene flame configuration for nickel and an air-nitrous oxide flame configuration for chromium. Burner parameters were set according to the instrument manufacturer’s recommendations. The test solutions were administered directly to the nebulizer, without prior dilution or mineralization. Instrument calibration was performed using the multi-element ICP Multi Element Standard Solution IV (1.11355 Supelco) in a matrix corresponding to the solutions used to extract the samples. The correctness of the calibration was verified using an independent control standard, Multi Element Standard Solution 5 for ICP (54704 Supleco). Absorbance was recorded in multiple modes, and the result was the average of a series of three measurements.

For each group, the amount of ions released per unit area of the incubated sample was determined. The calculations were performed as follows:

The mass *Xj* of the ions released since the beginning of the process was calculated from the following formula:

*Xj* = *Cj* × *Vc* (1)

where

- *Xj*—mass of the ions released from the sample [mg];
- *Cj*—spectrometrically determined ion concentration [mg/dm^3^];
- *Vc*—volume of the liquid in which the samples were submerged [dm^3^].

The mass of the ions released from the sample per surface unit was calculated from the following formula:

*Mj* = *Xj*/*Sp* (2)

where

- *Mj*—mass of the released ions per surface unit [mg/mm^2^];
- *Xj*—mass of the ions released from the sample [mg];
- *Sp*—sample surface in contact with the liquid [mm^2^].

Statistical analysis was performed using non-parametric methods to accommodate the multi-factorial design of the study (encompassing six sample groups, three immersion environments, three exposure periods, and two released ions). To evaluate global differences across the multiple sample groups within each environment and time point, the Kruskal–Wallis test was first applied. Subsequently, to determine specific differences between independent groups and identify where significant variations occurred, the Mann–Whitney U test was utilized as a post hoc procedure. To address the issue of multiple comparisons and control the family-wise error rate (Type I error), the Bonferroni correction was formally applied to adjust all calculated *p*-values. Statistical analyses were conducted using PQStat statistical software (version 1.6.4.122). [22]. The significance level was set at α = 0.05.

## 3. Results

**Figure 2:**
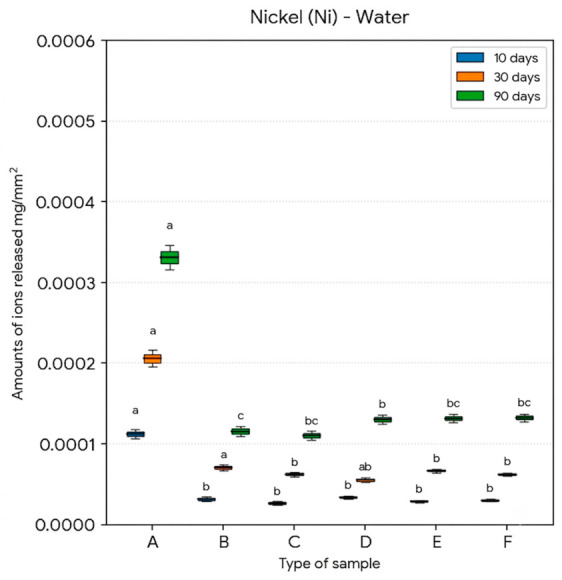
The cumulative release of nickel (Ni) ions into distilled water was quantified for each specific coating after 10, 30, and 90 days of exposure.

**Figure 3:**
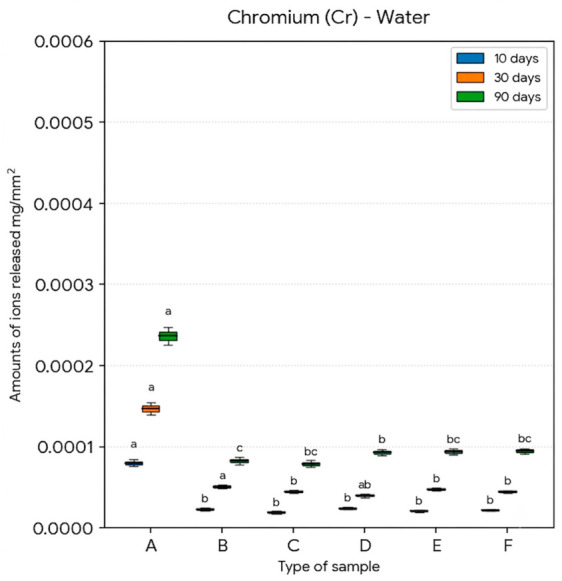
The cumulative release of nickel (Cr) ions into distilled water was quantified for each specific coating after 10, 30, and 90 days of exposure.

**Figure 4:**
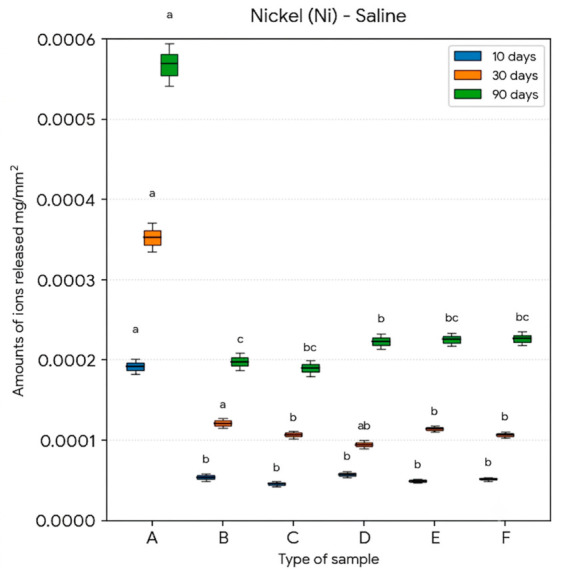
The cumulative release of nickel (Ni) ions into NaCl was quantified for each specific coating after 10, 30, and 90 days of exposure.

**Figure 5:**
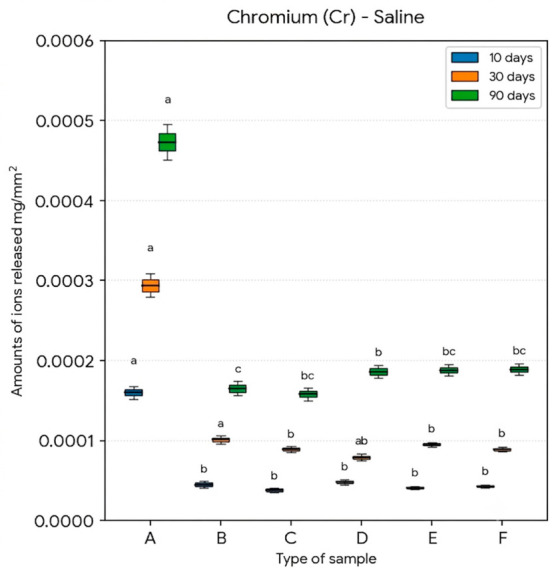
The cumulative release of nickel (Cr) ions into NaCl was quantified for each specific coating after 10, 30, and 90 days of exposure.

**Figure 6:**
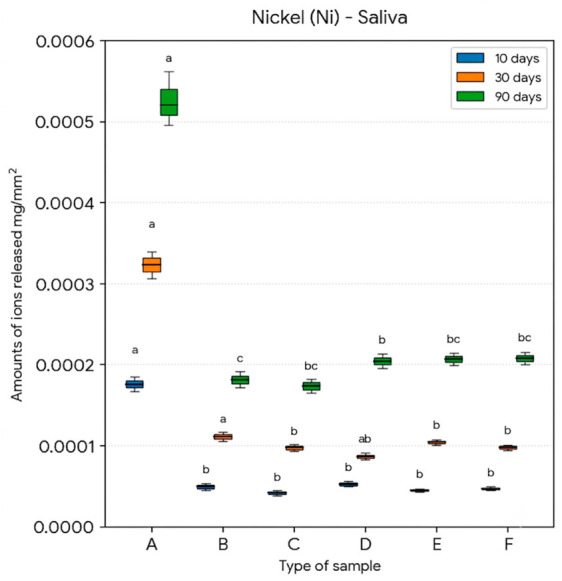
The cumulative release of nickel (Ni) ions into saliva was quantified for each specific coating after 10, 30, and 90 days of exposure.

**Figure 7:**
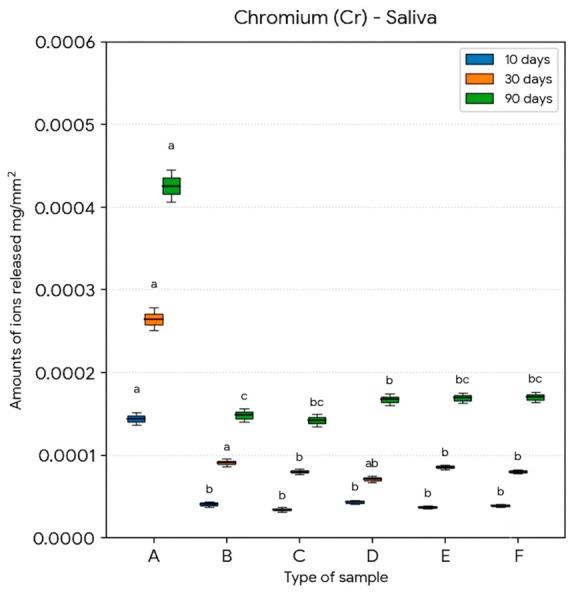
The cumulative release of nickel (Cr) ions into saliva was quantified for each specific coating after 10, 30, and 90 days of exposure.

The amounts of released ions from individual samples are shown in Figure 2, Figure 3, Figure 4, Figure 5, Figure 6 and Figure 7. To ensure the figures are fully self-explanatory and maintain clear statistical annotations, we have explicitly defined the meaning of the letter markers within the figure caption. The lower-case letters (a, b, bc, c) represent statistically homogeneous groups based on the non-parametric post hoc Mann–Whitney.

The graphs presented in Figure 2, Figure 3, Figure 4, Figure 5, Figure 6 and Figure 7 show that in each case, the amount of released ions in the uncoated group increased approximately twice after 30 days and approximately three times after 90 days compared to the group incubated for 10 days. For the coated samples, the ion release dynamics were as follows:

### 3.1. Distilled Water Environment

In the samples incubated in a distilled water environment, in each case in the samples covered with Si(C,N)-type coatings, a significant reduction in the number of ions in the solution was observed compared to the sample without coating. In the case of nickel ions, their amount decreased fourfold after 10 days and about threefold after 30 and 90 days. No significant differences were observed between the amounts of released ions in the coated samples. For chromium ions, after 10 days, the number of ions decreased from five (sample E) to ten (sample B) times. The amounts of released ions were similar after 30 days. The largest, ten-fold decrease was recorded in sample E, and the smallest, approximately five-fold, in sample C. Also in this case, no significant differences were found between the groups covered with coatings. For samples incubated for 90 days, the decrease in the amount of released ions was similar for all groups and was approximately 3-fold.

### 3.2. 0.9% NaCl Environment

In the samples incubated in a 0.9% NaCl environment after 10 days, a significant reduction in the amount of released nickel ions was observed in the samples coated with the assessed coatings in relation to the uncoated Ni-Cr alloy (A group), from about 7 times for the sample from group E to about 10 times for the remaining groups. In the case of the amount of chromium ions, the decrease was similar in all samples and was several-fold. After 30 days of incubation in a 0.9% NaCl environment, an approximately three-fold decrease in the amount of released nickel ions was observed in the samples from groups B and C, while in the remaining groups, the decrease was approximately five-fold. However, in the case of chromium ions, the decrease was about five times with respect to the uncoated Ni-Cr alloy. The amount of released chromium ions in all coated groups was similar. After ninety days of incubation in a 0.9% NaCl environment, the decrease in the amount of nickel ions compared to the uncoated sample was approximately three-fold in the samples from groups B and E and approximately two-fold in the remaining groups. As for chromium ions, it was similar in all samples and was approximately 5 times higher compared to the starting group.

### 3.3. Artificial Saliva Environment

In the artificial saliva environment, after 10 days, a significant reduction in the amount of released nickel and chromium ions was observed in the samples coated with the assessed coatings compared to the uncoated Ni-Cr alloy (group A). In the case of nickel ions for samples B, C, and D, an approximately thirteen-fold reduction was observed, while for groups E and F, it was approximately ten-fold. In the case of chromium ions, the largest decrease, approximately thirty-fold, was observed in group E, while the smallest, approximately twenty-fold, was in group B. The remaining samples fell between these groups. After thirty days of incubation in artificial saliva, an approximately six-fold decrease in the amount of released nickel ions and an approximately ten-fold decrease in the amount of chromium ions were noted. The amounts of released chromium ions in individual groups were similar. After incubation for 90 days, the amount of released nickel ions from all groups covered with coatings was similar and was approximately three times smaller than the amount of nickel ions released from the uncoated sample. As for chromium, the amount of released ions was about ten times smaller, and here too no significant differences were noted between the coated samples.

## 4. Discussion

Ceramic, metal alloy, polymer or composite discs are used to produce biomedical elements. Despite the wide availability of materials in medicine and dentistry in both digital and analog technologies, they still need to be modified. Improving materials is necessary to significantly improve patient comfort. Especially when we are increasingly seeing patients struggling with an excessive immune system response to common allergens [23]. Allergic reactions to metal alloys used in dental and orthodontic prostheses are becoming more and more common.

In such situations, a key role is played by, among others, a diagnostic interview and a medical questionnaire completed by a doctor. Therefore, an attempt was made to modify and improve the currently used dental alloys. As a solution, Si(C,N) coatings were used on the surface of the prosthetic alloy. A chromium-nickel-based alloy was used in this study. The research was focused on assessing the barrier potential of Si(C,N) coatings on the surface of a dental alloy. The null hypothesis of this study was rejected because Si(C,N) coatings affect ion release.

The ion release process is primarily driven by electrochemical corrosion in aggressive environments, such as saliva and body fluids, and is further accelerated by tribocorrosion. When articulating surfaces move against each other, the protective passive layer is mechanically removed, exposing the underlying metal and causing an intense release of ions. Additionally, this passive layer can be chemically degraded by the harsh environment of the oral cavity. This degradation is heavily influenced by factors such as fluctuating pH levels, temperature variations during meals, and the presence of bacteria.

The tests carried out showed that for each Si(C,N)-type coating, the number of ions released into individual liquids was significantly lower than in the case of samples without a protective coating. Statistical analysis showed that these differences were statistically significant (*p* < 0.05). No statistically significant differences were found between the coatings. Considering that the coatings have similar thickness, structure and corrosion resistance, such a result was to be expected [21,24]. Analysis of the results shows that the amount of nickel ions released into the solution is greater than the amount of chromium ions released. It appears that the primary cause of this result is the different content of these elements in the tested alloy (there is more than half as much chromium as nickel). Another reason may be, as explained by the authors of [19], the difference in the sizes of individual ions. Chromium ions, being larger, may have difficulty diffusing through the coating.

The use of Si(C,N)-type coatings to cover metal elements in contact with human body tissues may contribute to reducing adverse allergic responses. This is particularly important in dental prosthetics, where most prosthetic restorations are made of metal alloys. As we know, one of the main causes of contact allergies is metal ions released into the surrounding tissues, so creating a barrier that limits their passage will have positive effects. The release of metal ions from alloys is caused by corrosion phenomena. It should be remembered that the oral cavity is an aggressive environment for prosthetic elements made of metal alloys and others. In the oral cavity, metallic materials are subjected to various mechanical and chemical factors [25,26]. The severity of these harmful effects is directly related to the corrosion resistance of the alloy [27,28,29].

The tested coatings, as shown in [30], significantly reduce corrosion, so the coating’s action plays a double role. It is also worth mentioning the limited possibility of microorganism colonization on Si(C,N)-type coatings [31]. Beyond determining the most effective antibacterial coating, ensuring excellent biochemical and biomechanical compatibility is crucial for medical and dental biomaterials. Such tissue compatibility is essential to guarantee that the implanted material does not trigger any adverse or negative systemic reactions. A significant reduction in the number of adhered *E. coli* bacteria and *C. albicans* fungi was observed on coated samples compared to uncoated ones. Limiting the presence of microorganisms in the oral cavity on prosthetic elements is beneficial because they can contribute to the elimination of periodontal disease and extend the durability of implants [32,33,34].

In order to provide a comprehensive discussion of the individual layers, the characteristics of each layer were presented (Table 2). The information contained in the table comes from our previous studies [21,24].

Surface assessment is related to antibacterial activity. The best antibacterial effect is achieved by appropriately matching the wetting angle (hydrophobicity) to the antibacterial mechanism. The layers we obtained are characterized by water contact angle values (65–87 degrees). Although the water contact angles indicate that these coatings are hydrophilic, the values approach the critical angle of 90°. This is important for potential microbial colonization of the surface. The lower the wettability, the smaller the water-surface contact area, and therefore the lower the likelihood of colonization and biofilm formation. The surface analysis data also showed that the coatings we deposited had a smoother surface compared to the surface of the alloy used. However, the differences in roughness are not very large. It should be noted that with such thin coatings, they largely reproduce the roughness of the substrate. The degree of surface roughness is particularly important in the case of bacterial or fungal colonization. This is an important aspect in medical applications where antibacterial properties are desired. However, it should be remembered that the treatment with bone implants requires a much higher roughness value. The obtained values of the modulus of elasticity for the individual layers are satisfactory. This indicates that delamination will not occur in the future during the operation of the prosthetic restoration. The obtained coating thicknesses are sufficient to prevent delamination during occlusal stresses in the oral cavity. This is important because durability is required of prosthetic elements. Prosthetic materials should also be abrasion-resistant. Therefore, it is important that the substrate is not exposed and that the coating retains its protective properties during use. The obtained hardness values indicate that the applied silicon carbide nitride coatings will be able to protect the substrate material against abrasion. To accurately and completely determine wear resistance, tribological tests are necessary.

The observed ion release behavior directly correlates with the physical and mechanical properties of the Si(C,N) coatings. The barrier effect and the limitation of ion release may be related to the thickness of the sputtered layers, which effectively protects the Ni-Cr alloy substrate. The physical barrier is further enhanced by the low surface roughness of the coatings. Protects the surface against electrochemical effects caused by the aggressive oral environment. Additionally, chemical protection is related to the surface wettability and mechanical resistance of the layers. Surface properties such as (hydrophobicity/hydrophilicity) influence the interaction with the electrolyte at the external phase boundary, preventing deep penetration. Moreover, the high hardness and appropriately selected modulus of elasticity of the Si(C,N) matrix ensure high structural integrity. The mechanical resistance of the layer prevents it from cracking in the oral cavity environment, which ensures its long-term tightness and effectively inhibits the diffusion of ions.

The conducted research shows that there are no differences in the amount of ions released into the tested solutions by individual groups of samples. Although the statistical analysis showed no significant differences, these results should be interpreted with caution due to the limited sample size. This means that their diffusion-blocking properties are similar, and it is impossible to determine which coating is the most advantageous. Therefore, when selecting them in clinical procedures, other properties resulting from the application and operating conditions of the coated element should also be taken into account. As previous studies have shown, they show differences in hardness and modulus of elasticity.

The application of dental coatings relies on establishing both a physical and chemical barrier. The protective efficiency of these coatings can be attributed to several key mechanisms. Specifically, silicon carbonitride-based coatings exhibit a dense, amorphous structure that acts as a physical barrier, preventing the migration of metal ions from the prosthetic substrate into the patient’s body. Furthermore, these layers are characterized by strong covalent bonds (Si-C, Si-N); because the ionic radii of the substrate metal ions are too large, they cannot penetrate this molecular network, effectively hindering diffusion. Previous studies confirmed that the coating was defect-free, thereby eliminating potential pathways for metal ions to reach the surrounding environment and ensuring robust protection [21].

Covering prosthetic elements made of various metal alloys with coatings can significantly reduce the cytotoxicity of these elements. This allows them to be used in contact with human tissues. Current literature shows positive results from the use of various coatings. Among other things, a reduced level of toxicity is demonstrated by the authors Hank Won et al. [35]. They also studied nitride and carbide coatings. The material was coated with TiN and 3-1-2,5-diphenyl tetrazolium bromide (MTT) coatings [35]. In tests conducted by Brama et al. [36], titanium was coated with titanium carbide TiC using pulsed laser deposition (PLD) technology. The creators of this coating concluded that the positive effect stems from multiple factors, including the coating’s chemical composition and morphology [36]. In the publication by Petr Thomas et al. [37], the titanium nitride/zirconium nitride (TiN/ZrN) coated surface of cobalt–chromium–molybdenum alloy (CoCrMo) may cause an allergy. The research analysis included absorption spectrometry of released nickel, cobalt and chromium from the discs after exposure to distilled water and culture medium. The results showed that both coatings reduced the release of nickel and chromium from stainless steel and CoCrMo discs [37]. In order to effectively treat degenerative joint disease, the authors Ana Laura Puente Reyna et al. analyzed a metal ion-releasing barrier coating in a zirconium nitride (ZrN)-coated knee implant. The results of these analyses reveal that the release of metal ions from coated implants was reduced by up to three orders of magnitude compared to uncoated implants [38].

The conducted scientific research revealed valuable information on the protective effectiveness and ion release mechanisms of Si(C,N) coatings; however, several limitations should be taken into account. One of them is the fact that the evaluation was carried out strictly in vitro. Although artificial saliva and temperature fluctuations simulate some oral conditions, they cannot fully replicate the biological environment. In particular, the formation of complex biofilm and enzymatic activity. The studies are characterized by a relatively small sample size, which limits the statistical power of the analyses and may obscure subtle differences between the tested coating variants. This work focused mainly on the physicochemical and electrochemical properties of Si(C,N) layers. Biological studies, such as cytotoxicity and biocompatibility tests, were not performed. Future studies should include larger sample groups, extended immersion periods, and comprehensive in vitro cell culture models, which are required to further validate these coatings for clinical dental applications.

In summary, sputtered Si(C,N) coatings on dental alloy surfaces function effectively as a protective barrier, as demonstrated by the significantly reduced ion release compared to uncoated substrates. Consequently, these coatings could potentially be considered for future applications in dental prosthetics and implant components, provided that further in vivo studies confirm their biocompatibility.

## 5. Conclusions

1. Based on the conducted tests, it is concluded that all tested Si(C,N) coatings significantly limit the amount of metal ions in the surrounding medium.
2. Si(C,N)-type coatings can be considered for biomedical applications as protective coatings for base metal alloys.
3. Although no statistically significant differences in the number of released ions were detected among the individual coatings—which may be influenced by the limited sample size and statistical power—other properties relevant to the operating conditions of the components should also be considered during selection.

## Figures and Tables

**Table 1:** Designation and composition of samples [21].

Specimen Designation	Element						
	Si		N		C		at. C/N
	at. [%]	wt. [%]	at. [%]	wt. [%]	at. [%]	wt. [%]	
A	-	-	-	-	-	-	-
B	24.8	38.5	-	-	75.2	61.5	-
C	29.6	46.7	15.9	12.5	54.5	40.8	3.4
D	35.2	53.3	25.2	19.0	39.6	27.7	1.6
E	42.9	61.0	35.3	25.0	21.8	14.0	0.6
F	47.7	64.7	52.3	35.3	-	-	-

**Table 2:** Characteristics of Si(C,N) coatings [21,24].

Type of Sample	Layer Thickness [µm]	Coating Hardness [GPa]	Modulus of Elasticity of [GPa]	Surface Roughness Ra [µm]	Water Contact Angle [deg]
A	-	-	-	0.29 ± 0.3	81.5 ± 0.6
B	2.24 ± 0.09	13	130	0.070 ± 0.035	74.7 ± 4.5
C	2.59 ± 0.09	15	160	0.055 ± 0.009	86.8 ± 2.0
D	2.32 ± 0.05	12	130	0.066 ± 0.027	76.4 ± 1.9
E	1.95 ± 0.04	14	150	0.058 ± 0.06	75.2 ± 3.2
F	2.23 ± 0.03	14	170	0.044 ± 0.021	65.4 ± 0.8

## Data Availability

The original contributions presented in this study are included in the article. Further inquiries can be directed to the corresponding author.
